# Prostaglandin E_2_ production in the brainstem parabrachial nucleus facilitates the febrile response

**DOI:** 10.1080/23328940.2024.2401674

**Published:** 2024-09-24

**Authors:** Anders Blomqvist

**Affiliations:** Division of Neurobiology, Department of Biomedical and Clinical Sciences, Linköping University, Linköping, Sweden

**Keywords:** Fever, PGE_2_, EP_3_ receptors, median preoptic nucleus, thermosensory pathways

## Abstract

Our body temperature is normally kept within a narrow range of 1°C. For example, if our body temperature rises, such as in a hot environment or due to strenuous exercise, our thermoregulatory system will trigger a powerful heat defense response with vasodilation, sweating, and lowered metabolism. During fever, which often involves body temperatures of up to 41°C, this heat defense mechanism is apparently inhibited; otherwise, the rising body temperature would be immediately combated, and fever would not be allowed to develop. New evidence suggests how and where this inhibition takes place. In two consecutive studies from Cheng et al. and Xu et al., it has been shown that prostaglandin E_2_, which generates fever by acting on thermosensory neurons in the preoptic hypothalamus, also acts on neurons in the brainstem parabrachial nucleus, which receive temperature information from temperature-activated spinal cord neurons and relay this information to the thermoregulatory center in the hypothalamus to either induce cold or heat defenses. By acting on the same type of prostaglandin E_2_ receptor that is critical for fever generation in the preoptic hypothalamus, the EP_3_ receptor, prostaglandin E_2_ inhibits the signaling of the heat-responsive parabrachial neurons, while stimulating the cold-responsive neurons. These novel findings thus show that prostaglandin E_2_, by binding to the same receptor subtype in the parabrachial nucleus as in the preoptic hypothalamus, adjusts the sensitivity of the thermosensory system in a coordinated manner to allow the development of febrile body temperatures.

Fever is a cardinal symptom of infection and inflammation, and most likely adaptive. The elevated body temperature is considered to boost the immune response [1], and although antipyretic drugs are commonly used and prescribed, fever reduction may not be beneficial. Both clinical and preclinical studies have shown increased mortality when fever is suppressed with antipyretics [2–4].

It is well established that prostaglandin (PG) E_2_ is the final mediator of inflammation-induced fever [5–7]. PGE_2_ is synthesized by the inducible enzymes cyclooxygenase (COX)-2 and microsomal prostaglandin E synthase-1 (mPGES-1) [8,9]. Over-the-counter antipyretic drugs inhibit COX-2, as was demonstrated by John Vane and his collaborators in the early 1970s [10,11]. In the brain, PGE_2_ is synthesized by vascular endothelial cells [12–14] that have been shown to express receptors for pyrogenic cytokines, such as interleukin (IL)-1 and IL-6 [15,16]. Genetic deletion or silencing in brain endothelial cells of the IL-1 and IL-6 receptors (IL-1 type 1 receptor and IL-6 receptor α) or their downstream signaling molecules attenuates or abolishes the fever by attenuating the PGE_2_ synthesis [16–19], as does endothelial deletion of COX-2 or mPGES-1 [14,20–22]. PGE_2_ exerts its fever-generating effect by binding to PGE_2_ EP_3_ receptors [23], the critical site being EP_3_ receptor expressing neurons in or in the immediate vicinity of the median preoptic nucleus (MnPO) (Figure 1), as shown by studies in which EP_3_ receptors were selectively deleted in this nucleus [24]. The attenuation of the febrile response to an intraperitoneal injection of lipopolysaccharide, a commonly used model for eliciting fever in experimental animals [25], was linearly correlated with the extent of the EP_3_ receptor deletion in the MnPO, but not with that in adjacent nuclei. In the same vein, microinjection of PGE_2_ caused fever when localized to or in the immediate vicinity of the MnPO but not to more distant sites [26], and microinjections of a COX inhibitor into the MnPO attenuated LPS-induced fever, whereas such injections at other sites did not [27]. Similarly, virus-mediated restoration of mPGES-1, localized to the MnPO, resulted in a temperature elevation in response to LPS in otherwise fever refractive mPGES-1 knockout mice [28].

**Figure 1.**
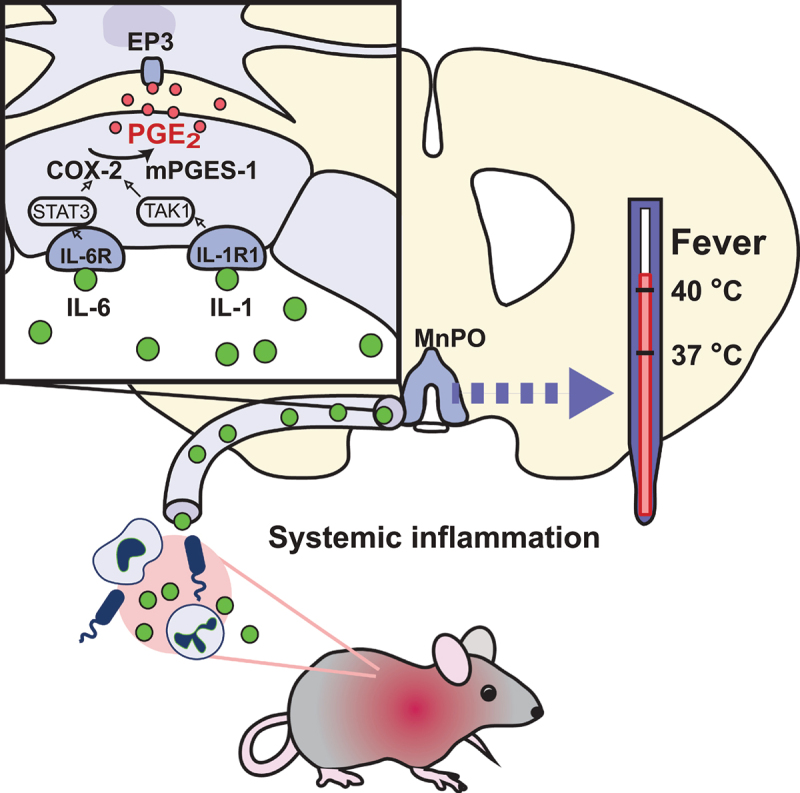
Mechanisms of inflammation-induced fever. During systemic inflammation, cytokines, such as IL-1 and IL-6, are released into the circulation. The binding of these cytokines to their receptors (IL-1R1, IL-6R) on brain endothelial cells induces via the NFκB (TAK1) and STAT3 pathways the expression of COX-2 and mPGES-1, resulting in the production of PGE_2_ that is released into the brain parenchyma where it by binding to EP_3_ receptors in the median preoptic nucleus (MnPO) evokes thermogenesis. Adapted from [14].

The EP_3_ receptor expressing neurons in the MnPO have been reported to be inhibited by PGE_2_ [29–31], resulting in the release of downstream thermogenic pathways that originate in the dorsomedial hypothalamic nucleus/dorsal hypothalamic area and the raphe pallidus nucleus. The EP_3_ expressing neurons, which also express the neuropeptide pituitary adenylate cyclase-activating polypeptide [31,32], have been identified as GABAergic in rats [33,34], and as glutamatergic in mice [35], however in both cases they are suggested to exert their fever generating action by disinhibition of their downstream targets [34,35].

While the PGE_2_ synthesis and its binding to EP_3_ receptors in the MnPO is critical for the fever response, during inflammation PGE_2_ is synthesized throughout the brain [28,36–38] and EP_3_ receptors are also widely distributed [39,40]. In addition to fever, the EP_3_ receptors have been implicated in hyperalgesia and stress hormone release [6], but the functional role of much of the extensive PGE_2_ production and EP_3_ expression is largely obscure. However, quite recently, the significance of the dense EP_3_ receptor expression in the brain stem parabrachial nucleus [41] (Figure 2) was elucidated. In a paper from the laboratory of Jie Zhang, Chengdu, China [43], it was shown that microinjection of a cyclooxygenase inhibitor into the lateral parabrachial nucleus of rats attenuated LPS-induced fever, and that direct PGE_2_ administration into this nucleus resulted in elevated body temperature. In a subsequent follow-up study [44], some of the same authors demonstrated that PGE_2_ activated MnPO projecting neurons in the external lateral parabrachial subnucleus while at the same time inhibiting MnPO projecting neurons in the dorsal lateral subnucleus. They further showed that selective genetic lesioning or inhibition of the pathway from the parabrachial nucleus to the MnPO attenuated the increased body temperature induced by local injection of PGE_2_ into the parabrachial nucleus, and that microinjection of an EP_3_ receptor agonist mimicked the pyrogenic action of the PGE_2_, whereas microinjection of an EP_3_ receptor antagonist attenuated the PGE_2_ elicited response.

**Figure 2.**
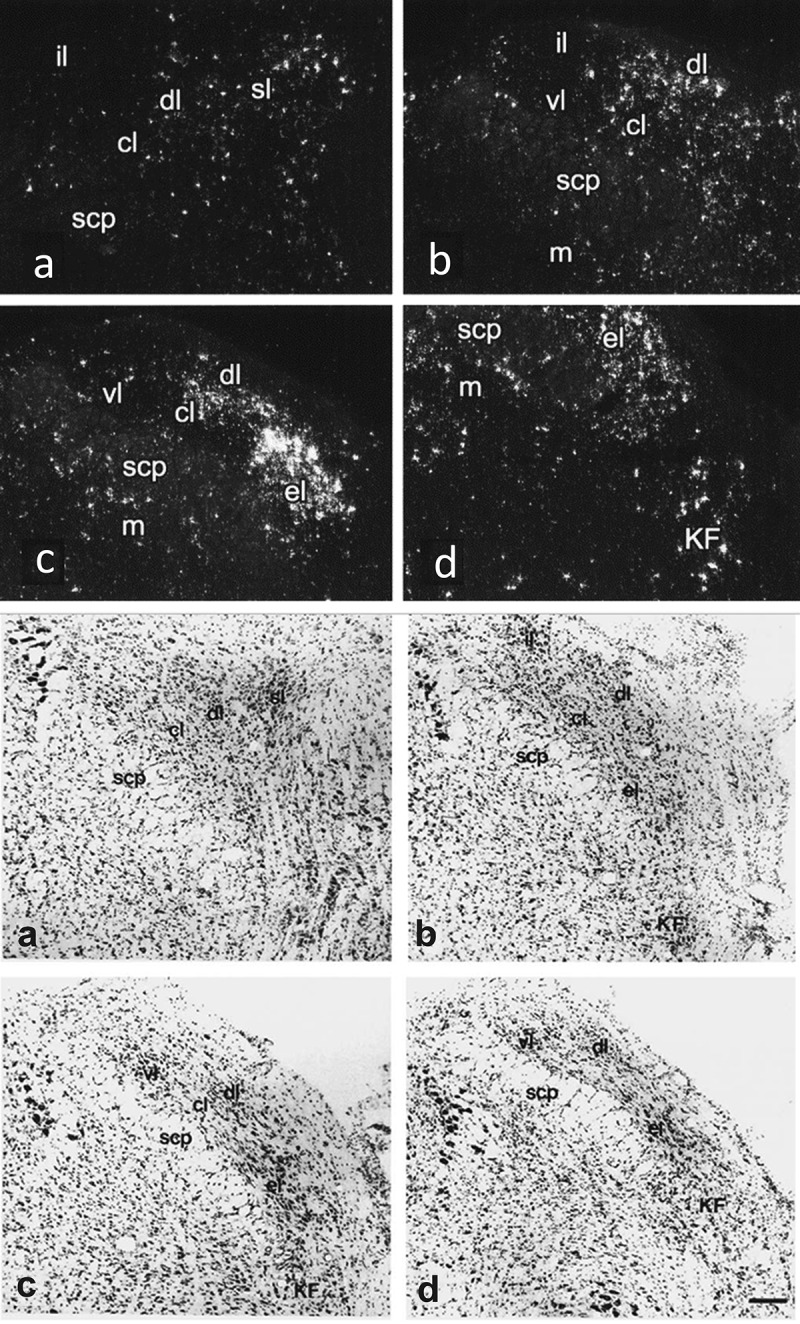
Top: Radio-labeled in situ hybridization showing EP_3_ receptor expression in the parabrachial nucleus of the rat. a–d are frontal sections ordered from rostral to caudal. cl, central lateral nucleus; dl, dorsal lateral nucleus; el, external lateral nucleus; il, internal lateral nucleus; KF, Kölliker–fuse nucleus; m, medial parabrachial nucleus; scp, superior cerebellar peduncle; sl, superior lateral nucleus; vl, ventral lateral nucleus. Adapted from [41]. Bottom: The cytoarchitecture of the different parabrachial subnuclei is shown in thionin-stained sections (taken from [42]) that are approximately from the same rostrocaudal levels as those showing the EP_3_ receptor expression. Sections are separated by 140 μm, with section b being at the level of separation of the inferior colliculus from the pons. Scale bar = 100 μm but note that magnification in the top dark-field micrographs is about two times higher.

The authors interpret their findings to suggest that PGE_2_ produced locally in the lateral parabrachial nucleus and acting there on EP_3_ receptors is an additional mechanism for fever to PGE_2_ synthesis and EP_3_ receptor binding in the MnPO. I will here provide a somewhat different perspective: I propose that the PGE_2_ synthesized in the parabrachial nucleus is not febrile in itself but works in concert with the fever generating PGE_2_ in the MnPO to adjust the sensitivity of the thermosensory system to the febrile body temperature. My argument is based on the fact that, as discussed above, without EP_3_ receptors in the MnPO there is no fever [24], irrespective of the presence of induced PGE_2_ production throughout the brain and of abundance of EP_3_ receptors at many sites in addition to MnPO.

In groundbreaking studies some 15 years ago, Shaun Morrison and Kazuhiro Nakamura showed that thermosensory information from the body, transmitted by neurons located in the superficial lamina of the spinal dorsal horn, was relayed to the thermoregulatory center in the preoptic hypothalamus via neurons located in the lateral parabrachial nucleus [45,46]. The warm and cool-activated parabrachial neurons were distinct, with those responding to cooling reported to be located to the external lateral parabrachial subnucleus [45], and those responding to warming located to the dorsal lateral parabrachial subnucleus [46]. Subsequent studies have shown that the parabrachial neurons activated by cooling are not located to the external lateral subnucleus proper, but in a region slightly rostral to it, named the rostral-to-external lateral subnucleus [47].

The projection to the preoptic hypothalamus from the warm-activated neurons in the dorsal lateral parabrachial subnucleus is best characterized. It is a pathway for heat-defense: It projects to preoptic neurons involved in regulating body temperature, eliciting heat-dissipating mechanisms, such as cutaneous vasodilation and, in humans, sweating, as well as cold seeking behavior [48]. The warm-activated parabrachial neurons have been shown to use glutamate as neurotransmitter [49], but a large proportion of them also are opioidergic, expressing dynorphin [47,49–51], and photostimulation of the dynorphinergic terminals in the preoptic hypothalamus as well as chemogenetic activation of the preoptic projecting dynorphin neurons elicited hypothermia [49,51]. While it has not yet been shown that the endogenous release of dynorphin by these neurons contributes to the hypothermia, such an effect is suggested by the finding that κ-opioid receptor agonists microinjected into the preoptic hypothalamus lower the body temperature [52]. Notably, a large proportion of the dynorphinergic cells in the dorsal lateral subnucleus express EP_3_ receptors [53] (Figure 3), suggesting that they are prostaglandin sensitive. Some of the preoptic projecting neurons express cholecystokinin. These cells, which either are a subset of the dynorphinergic neurons [49], or constitute a separate population [51], have been reported to specifically elicit vasodilation [51]. Recently, it has also been reported that the dynorphinergic neurons in the dorsal lateral parabrachial subnucleus project directly to the dorsomedial hypothalamus (DMH), there to inhibit thermogenesis-promoting neurons [54] (Figure 4).

**Figure 3.**
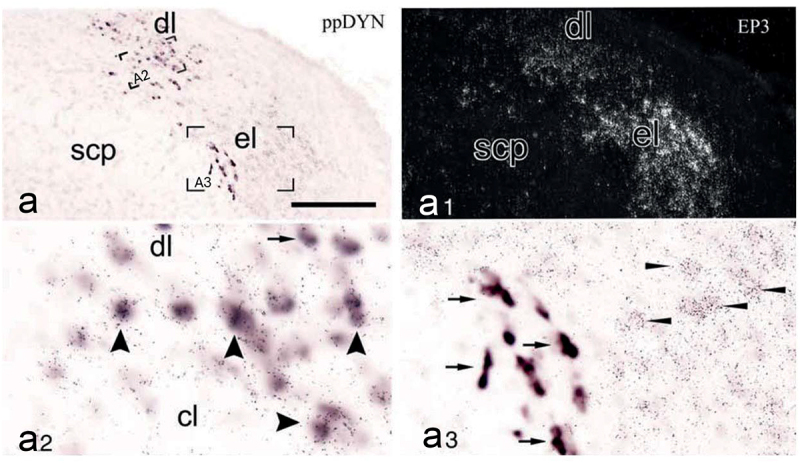
Co-expression of digoxigenin labeled preprodynorpin (ppDYN) mRNA (a) and radiolabeled EP_3_ receptor mRNA (a_1_) in the lateral parabrachial nucleus of the rat. The dynorphin neurons in the dorsal lateral subnucleus (dl, a_2_) display extensive double labeling with EP_3_ (wide arrowheads). Another ppDYN expressing population, located in the inner part of the external lateral subnucleus (el, a_3_), is single labeled. cl, central lateral subnucleus; scp, superior cerebral peduncle. Adapted from [53].

**Figure 4.**
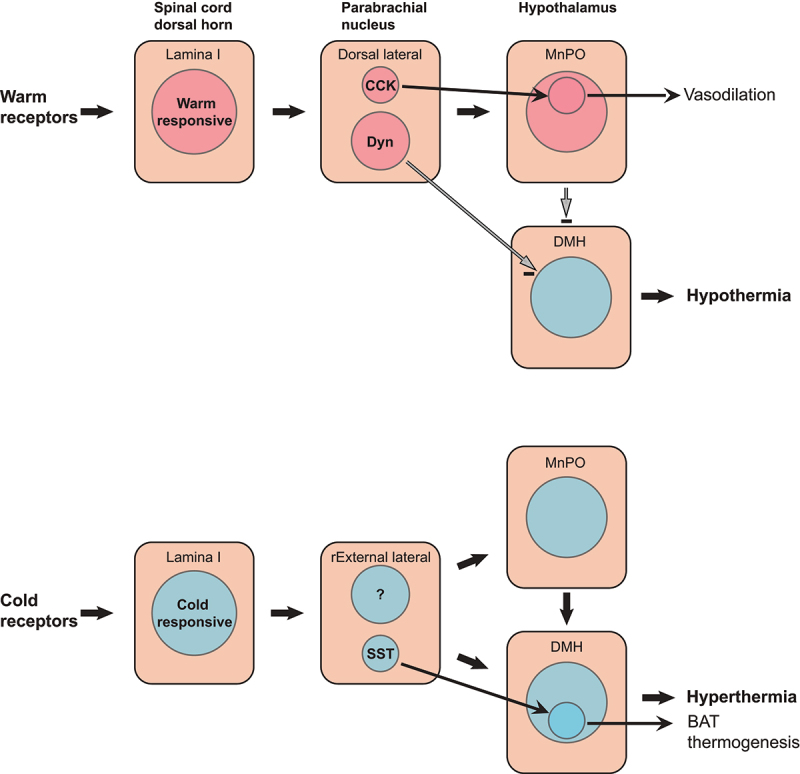
The thermoregulatory spino-parabrachial-hypothalamic pathways. Warm-responsive spinal dorsal horn neurons activate median preoptic (MnPO)-projecting dynorphinergic (Dyn) and cholecytokininergic (CCK) neurons in the dorsal lateral parabrachial subnucleus to evoke a cold defense response, with the CCK neurons specifically being involved in vasodilation. The dynorphinergic neurons also directly project to the dorsomedial hypothalamus (DMH), where they inhibit thermogenesis-promoting neurons. Cold-responsive dorsal horn neurons activate MnPO and DMH projecting neurons in the rostral-to-external (rExternal) lateral subnucleus, which evoke hyperthermia, with neurons expressing somatostatin (SST) being specifically involved in brown adipose tissue (BAT) thermogenesis.

The neurons in the rostral-to-external lateral parabrachial subnucleus are involved in cold defense. Their activation will elicit sympathetic and shivering thermogenic responses [48]. Like the warm-activated neurons, the neurons activated by cold project to the MnPO of the hypothalamus, however, this pathway alone does not seem to be sufficient for a robust cold defense, which requires the involvement of cold-activated neurons in the dorsomedial hypothalamus [55]. Recently, a direct projection to the dorsomedial hypothalamus from cold activated parabrachial neurons was described [56]. Activation of this pathway, which seems to work in parallel with the projection to the MnPO, induced strong cold-defense responses, including brown adipose tissue thermogenesis and muscle shivering [56]. Similar to the warm-activated neurons, the cold-activated neurons that project to the MnPO or to the ventromedial hypothalamus are glutamatergic [49,56]. A subset of the neurons projecting to the ventromedial hypothalamus express somatostatin, and this population was reported to specifically induce brown adipose tissue thermogenesis, but otherwise the genetic identity of the cold-activated neurons is not known [56] (Figure 4). Given that the activation of μ-opioid receptors in the preoptic hypothalamus induces hyperthermia [52], enkephalins, which are expressed by large numbers of neurons both within and around the external lateral subnucleus [57] that also co-express EP_3_ receptors [53], are potential candidates for mediating the cold-evoked responses.

What will happen to these two opposing thermoregulatory pathways during fever? When we are overheated due to a hot environment or as a consequence of strenuous exercise, warm receptor that are located in the skin, visceral organs, and the spinal cord [58] will activate warm-responsive spinal dorsal horn neurons that project to the dorsal lateral parabrachial subnucleus, whereas the cold-responding pathway will be silenced, hence eliciting a vigorous heat defense response with vasodilation and sweating (Figure 4). If the same heat defense mechanism would be evoked during infection, it would obviously counteract the febrile response. It is here the local PGE_2_ production and the EP_3_ receptors in the parabrachial nucleus are likely to come into play: When PGE_2_ is produced in the MnPO and increases the body temperature, PGE_2_ is concomitantly produced in the parabrachial nucleus, and this latter PGE_2_ production inhibits the heat defense pathway of the dorsal lateral subnucleus, while at the same time facilitating signal transduction in the cold defense pathway of the rostral-to-external lateral subnucleus (Figure 5). Consequently, if the PGE_2_ synthesis in the parabrachial nucleus is inhibited by local injection of a COX inhibitor, the fever response is indeed attenuated [43], and conversely, if the heat defense mechanism is blocked, the fever is augmented [51]. Hence, because of the PGE_2_ synthesis in parabrachial nucleus, the thermosensory pathways will no longer counteract the febrile response but act in synergy with it. It should be noted that because the PGE_2_ action is not on the thermosensory neurons in the spinal cord but on the relay in the parabrachial nucleus, the break on the heat defense pathway seems to occur without affecting our temperature perception, which is mediated by a spinothalamocortical pathway [59].

**Figure 5.**
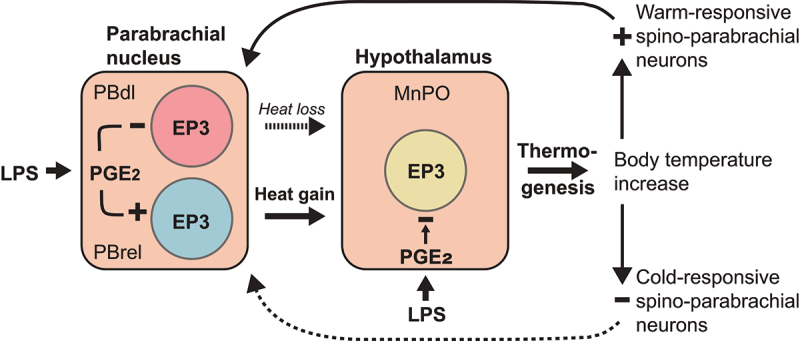
Concerted action of the thermogenic and afferent thermoregulatory systems in creating the febrile response. An inflammatory stimulus, such as lipopolysaccharide (LPS) results in the formation of PGE_2_ in the brain. The binding of PGE_2_ to EP_3_ receptors on neurons in the median preoptic nucleus (MnPO) inhibits these neurons, resulting in release of thermogenesis and subsequent rise in body temperature (fever). The increased body temperature will, while inhibiting cold-responsive thermoreceptive neurons, activate warm-responsive spino-parabrachial neurons that target heat defense eliciting neurons in dorsal lateral subnucleus of the parabrachial nucleus (PBdl). Their activation would hence counteract the PGE_2_ induced thermogenesis. However, the PGE_2_ produced in the parabrachial nucleus will through binding to EP_3_ receptors on the PBdl neurons inhibit these neurons, while at the same time activating the neurons in the rostral-to-external lateral subnucleus (PBrel) that are involved in the cold defense, hence creating a concerted action with the PGE_2_ released in the MnPO. Solid and dotted lines indicate activated and inhibited pathways, respectively.

The opposing effect of PGE_2_ on the warm- and cold-responsive parabrachial neurons, respectively, the warm-responsive being inhibited and the cold-responsive excited, may seem puzzling, because the EP_3_ receptor is mainly an inhibitory receptor. However, at least three EP_3_ receptor subtypes exist, EP3α, EP3β, and EP3γ [29], with the EP3α and EP3β subtypes being coupled to inhibitory G-proteins, hence being inhibitory, whereas the EP3γ subtype is coupled to stimulatory G-proteins and being excitatory. The presence of the EP3γ subtype among the cold-responsive parabrachial neurons is suggested by Xu et al. [44] to explain the excitatory action of PGE_2_ on these neurons.

By showing how PGE_2_ synthesis and EP_3_ receptors in the brain stem parabrachial nucleus influence the thermosensory processing, the studies by Cheng et al. and Xu et al. [43,44] have added an important piece of information on the complex mechanisms underlying the febrile response. However, while these studies have shown how PGE_2_ signaling in the parabrachial nucleus allows the development of febrile body temperatures, an important consequential question is how the prevention of overheating works during peripheral inflammation. With the heat-defense spino-parabrachial-preoptic pathway inhibited, warm-sensitive neurons (WSNs) in the preoptic hypothalamus that monitor the internal temperature [60–62] seem most likely to be critical in limiting the febrile response, but how these neurons relate to those receiving thermoreceptive information relayed via the parabrachial nucleus is largely unknown, as is the extent to which WSNs are identical to the EP_3_ expressing MnPO neurons [31] and therefore inhibited by the PGE_2_ production. Furthermore, in addition to MnPO and the parabrachial nucleus, EP_3_ receptors are heavily expressed in other parts of the temperature regulating system, such as the raphe pallidus nucleus, which is a critical hub in the descending thermogenic pathway [9,63]. How PGE_2_ signaling in that nucleus regulates the febrile response is yet another interesting question to be answered [64,65]. Finally, the findings discussed in this review should be considered with the caveat that almost all work has been performed in rodents. The extent to which the results are applicable to human physiology remains to be investigated.

## List of abbreviations

COX, cyclooxygenase

IL, interleukin

LPS, lipopolysaccharide

MnPO, median preoptic nucleus

mPGES, microsomal prostaglandin E synthase

PG, prostaglandin

WSNs, warm-sensitive neurons
